# Quantitative Extraction of the Self-Absorption Probability in Quantum Dot Color Conversion Films and Its Modulation by TiO_2_

**DOI:** 10.3390/nano16140842

**Published:** 2026-07-09

**Authors:** Kinza Batool, Youngji Lim, Kyoungwon Park, Bum-Joo Lee

**Affiliations:** 1 Department of Flexible and Printable Electronics, Jeonbuk National University, 567, Baekje-daero, Deokjin-gu, Jeonju-si 54896, Republic of Korea; batoolkinza0011@gmail.com; 2 Display Research Center, Korea Electronics Technology Institute (KETI), 25, Saenari-ro, Bundang-gu, Seongnam-si 13509, Republic of Korea; dudwl0519@keti.re.kr (Y.L.); kw1park@keti.re.kr (K.P.)

**Keywords:** quantum dots, color conversion film, self-absorption, reabsorption, photoluminescence quantum yield, titanium dioxide (TiO_2_), light scattering

## Abstract

Self-absorption limits efficiency and causes spectral redshift in quantum dot color conversion (QDCC) films, yet it is usually inferred indirectly rather than measured. In this study, we extract the self-absorption probability quantitatively from film photoluminescence (PL) spectra using the correction method of Ahn et al. The films span a wide thickness range at two quantum dot (QD) concentrations, with and without TiO_2_. The converted emission peak wavelength scales linearly with the extracted self-absorption probability. For a given TiO_2_ condition, this relation is independent of QD concentration. The self-absorption probability increases with both film thickness and QD concentration, reflecting longer optical path lengths and more reabsorption events. The intrinsic quantum yield from the same analysis is thickness-independent within each condition, supporting the extracted values. The addition of TiO_2_ increases blue light absorption and lowers the extracted self-absorption probability of the converted emission, consistent with enhanced light scattering. With TiO_2_, the remaining self-absorption is concentrated toward shorter wavelengths, producing a larger peak shift at comparable self-absorption probability. The external quantum efficiency increases with thickness at the lower concentration but not at the higher one, reflecting the competition between blue absorption and self-absorption.

## 1. Introduction

Quantum dots (QDs) have emerged as a key material for next-generation displays and optical devices owing to their size-tunable optical properties, narrow emission linewidths, and high photoluminescence quantum yield (PLQY) [1,2,3]. While conventional liquid crystal displays (LCDs) with phosphor-based backlights are typically limited to approximately 74% of the National Television System Committee (NTSC) color gamut, integrating QD technology has expanded this range to exceed 130%, achieving color purity comparable to or surpassing that of organic light-emitting diodes (OLEDs) [4,5,6].

Initially, QD displays were primarily commercialized as quantum dot enhancement films (QDEFs). However, their continued dependence on absorptive color filters results in significant optical loss, thereby limiting overall luminous efficiency [6]. To overcome this fundamental limitation, research focus has rapidly shifted toward quantum dot color conversion (QDCC) technologies. In QDCC architectures, QDs are patterned directly into each subpixel, enabling direct color conversion and offering significantly higher efficiency than QDEF-based approaches [7,8,9].

To fully realize the potential of QDCC, the incident excitation light must be efficiently converted into the desired emission wavelengths. The optical external quantum efficiency (EQE), defined as the number of emitted photons that escape the film per incident excitation photon, and the converted peak emission wavelength are therefore the primary determinants of QDCC performance. Experimentally, the optical EQE has been reported to exhibit a maximum at a specific combination of film thickness and QD concentration [10,11,12], while the converted peak wavelength shifts toward longer wavelengths (redshift) as the film thickens or the QD loading increases [8,12,13]. Both phenomena are commonly attributed to self-absorption arising from partial spectral overlap between the absorption and emission spectra of the QDs [8,14]. A photon emitted in this overlap region may be reabsorbed by a neighboring QD before escaping. In thicker or more concentrated films, this reabsorption loss eventually outweighs the additional conversion gain, which has been used to explain the EQE maximum observed at an intermediate thickness and concentration [10,12]. The same process drives the redshift. Because the absorption is stronger on the short-wavelength side than on the long-wavelength side, photons emitted on the short-wavelength side are preferentially reabsorbed and re-emitted according to the intrinsic line shape. The escaping spectrum is therefore systematically redshifted, and the shift increases with thickness and QD loading [14,15]. Self-absorption therefore governs both performance metrics, particularly in optically dense films, and a quantitative understanding of it is essential to the optical design of high-performance QDCC films.

The incorporation of high-refractive-index nanoparticles, particularly TiO_2_, into QD films has been widely investigated to address this limitation [7,11,12,15,16]. The addition of TiO_2_ simultaneously increases the external quantum efficiency and reduces residual blue light leakage at a given film thickness, with reported enhancements in conversion efficiency ranging from about 30% to over 80% [11,12,15,16]. It also lowers the film thickness required for maximum conversion, which is beneficial for high-resolution micro-light-emitting diode (micro-LED) displays where thick color conversion layers are difficult to pattern [12,15,16]. These gains are generally attributed to Mie scattering by submicron TiO_2_ particles. The large refractive-index contrast with the polymer host lengthens the effective path of the excitation light and increases its absorption by the QDs [11,16]. Scattering also redirects converted photons that would otherwise be confined by total internal reflection, improving light extraction [7,12].

Various analytical and numerical models have been developed to describe the optical behavior of QDCC films. Xu et al. introduced a dosage-factor framework in which the product of film thickness and QD concentration (*u* = *d*·*c*) serves as the governing variable for blue light transmittance and optical density, and the framework predicts a non-monotonic light-conversion efficiency with an analytically derived optimum [17]. Full-spectral models have further incorporated reabsorption and re-emission processes, demonstrating that self-absorption of QD-emitted photons is a dominant loss mechanism limiting the radiant efficacy of QD color-converted LEDs [14]. Numerical methods, including finite-difference time-domain (FDTD) [18], Monte Carlo ray tracing [19], and LightTools-based simulations [12], have also been employed to obtain the underlying optical parameters and to optimize film thickness, QD loading, and scattering-particle content in QDCC structures.

Across these modeling efforts, self-absorption is consistently identified as the dominant factor limiting conversion efficiency and shifting the emission spectrum of QDCC films [14,17]. Yet in most cases, self-absorption is estimated only indirectly, through indicators such as observed redshifts or the presence of an optimal thickness, rather than being measured as an independent quantity. To date, the self-absorption probability and its dependence on film thickness and QD loading have not been directly extracted from QDCC films, leaving a key parameter of color conversion physics characterized only through secondary observables.

The method for determining the self-absorption probability *a* and the intrinsic photoluminescence quantum yield QY_0_ was systematically established by Ahn et al. using perylene red dispersed in poly(methyl methacrylate) (PMMA) films [20]. As in optically dense QDCC films, perylene red films exhibit substantial overlap between absorption and emission, and thus emitted photons are reabsorbed before escaping, distorting the observed emission spectrum and quantum yield. The correction is based on the following principle. Reabsorption removes photons preferentially from the short-wavelength side of the emission, where absorption and emission overlap, but it leaves the shape of the long-wavelength tail essentially unchanged, since this region lies beyond the absorption edge. Its shape, therefore, matches that of a dilute reference free of self-absorption. By scaling the dilute-reference spectrum to match the film emission in this tail, one can reconstruct the spectrum the film would have emitted without self-absorption, and the difference between this reconstructed spectrum and the measured one gives the fraction of photons lost to reabsorption [20].

This approach builds on the normalization introduced by Birks for concentrated fluorescent solutions [21] (pp. 97–100), which Ahn et al. extended to solid-state films and integrating-sphere PLQY measurements. Correction strategies based on this method have since been applied to dye-doped PMMA films for luminescent solar concentrators [22], LED phosphor/silicone composites [23], conjugated polymer–inorganic hybrid films [24], and luminescent coordination-polymer composites [25], indicating its applicability to optically dense luminescent systems. Despite its conceptual simplicity and direct connection to the underlying photophysics, this approach has not yet been systematically applied to QDCC films, where the combined influence of high QD loading, film thickness, and TiO_2_ scattering on the self-absorption probability *a* remains insufficiently quantified.

In this work, we apply the self-absorption correction method of Ahn et al. to QDCC films and extract the self-absorption probability directly from their photoluminescence (PL) spectra. Films spanning a wide range of thickness at two QD concentrations, with and without TiO_2_, are examined. The CdSe/ZnSe/ZnS QDs were chosen because they offer a high quantum yield, a narrow emission linewidth, and stable, well-understood optical properties. These properties make them well suited for developing and validating the extraction method. The method itself does not depend on the emitter material. Using the extracted self-absorption probability, we interpret the redshift of the converted emission and the thickness dependence of the conversion efficiency, and identify how TiO_2_ modifies self-absorption. This establishes self-absorption as a directly measurable descriptor of QDCC films.

## 2. Materials and Methods

### 2.1. Sample Fabrication

Red CdSe/ZnSe/ZnS (core/shell/shell) QD ink was supplied by Fine Lab Co., Ltd. (Daejeon, Republic of Korea). Ethyl alcohol (99.9%), n-hexane (95.0%), propylene glycol monomethyl ether acetate (PGMEA, 99.0%), and toluene (99.5%) were purchased from Samchun Pure Chemical Co., Ltd. (Seoul, Republic of Korea). The dispersant BYK-111 (BYK-Chemie GmbH, Wesel, Germany), binder SSCQB230 (Uniam Co., Ltd., Seoul, Republic of Korea), and radical photoinitiator OXE-02 (BASF Schweiz AG, Basel, Switzerland) were used for ink formulation. A TiO_2_ nanoparticle dispersion (rutile, 200–300 nm, 30 wt% in propylene glycol monomethyl ether (PGME); Ditto Technology, Gunpo, Republic of Korea) was used as the scattering agent. Glass substrates were purchased from Chemtronics (Seongnam, Republic of Korea). All reagents were used as received without further purification.

The commercial CdSe/ZnSe/ZnS QD dispersion was first purified to remove residual ligands and organic impurities. Typically, 5 mL of the QD dispersion was mixed with 40 mL of ethanol and centrifuged at 12,000 rpm, which corresponds to about 14,600× *g*, for 10 min using a fixed angle rotor (Combi R515, A50c-6, *r* = 91 mm, Hanil, Gimpo, Republic of Korea) to precipitate the QDs. After discarding the supernatant, the precipitate was redispersed in 5 mL of toluene by ultrasonication for 10 min. This purification cycle was repeated to reduce residual ligands and organic impurities. Finally, the QDs were dried under vacuum for 1 h to yield purified QD powders.

To improve compatibility with the polymeric matrix, ligand exchange was performed on the purified QD powders using the BYK dispersant in PGMEA. The QDs, PGMEA, and BYK were mixed at a weight ratio of 1:20:1 and stirred at 500 rpm at 140 °C for 1 h. The ligand-exchanged QDs were precipitated by adding deionized water and collected via centrifugation to remove residual byproducts. In a second washing step, the QDs were redispersed in 5 mL of PGMEA by sonication, precipitated again with n-hexane, and centrifuged. The resulting QDs were finally dried under vacuum to obtain stable ligand-exchanged powders.

The ligand-exchanged QD powders were then formulated into photocurable inks for color conversion films. The QDs were combined with SSCQB230 binder resin and OXE-02 photoinitiator at solid weight ratios of QD:binder:photoinitiator = 20:79:1 for the 20 wt% ink and 40:59:1 for the 40 wt% ink. The 20 and 40 wt% QD loadings were chosen to span the practically relevant range, from partial blue absorption at 20 wt% to already high blue absorption at 40 wt%. For these TiO_2_-free inks, no scattering particles were incorporated. To ensure homogeneous dispersion, the mixtures were stirred at 350 rpm at 50 °C for 48 h, with PGMEA added as a solvent to adjust the viscosity for spin coating and to enhance QD dispersibility. In a separate set of inks, a 5 wt% TiO_2_ loading was added as a representative scatterer content, high enough to produce a clear scattering effect while keeping the film optically uniform. The two QD loadings and the two TiO_2_ conditions together form a 2 × 2 set that isolates the effect of QD concentration and the effect of scattering.

These TiO_2_-containing inks were prepared by adding the commercial TiO_2_ dispersion described above to the 20 wt% and 40 wt% QD inks at the calculated amount to reach 5 wt% TiO_2_ relative to total solids. The mixtures were further stirred at 350 rpm and 50 °C to ensure uniform dispersion of the TiO_2_ particles throughout the ink. In both formulations, 0.45 g of PGMEA was added as a carrier solvent to adjust the ink for spin coating and to improve QD dispersion. The QD loading refers to the QD fraction of the nonvolatile solids. PGMEA leaves the film during spin coating and curing and does not enter the cured film composition.

QDCC films were fabricated by spin-coating the formulated inks onto glass substrates (2.5 × 2.5 cm^2^) that had been sequentially cleaned by ultrasonication in acetone, isopropanol, and deionized water for 10 min each. Film thickness was controlled by adjusting the spin speed. Spin speeds ranging from 1000 to 8000 rpm yielded film thicknesses of 2.69–11.4 µm for the 20 wt% ink and 3.5–14.1 µm for the 40 wt% ink, covering the optically dense regime relevant to QDCC operation. The film thickness obtained at each spin speed for all conditions is listed in Table S1. The TiO_2_-containing inks were deposited under the same spin-coating conditions and spanned a similar thickness range. Following deposition, the films were cured under UV irradiation for 10 s to ensure crosslinking and structural stability.

### 2.2. Characterization

The optical properties of the QD inks and the fabricated QDCC films were characterized by ultraviolet–visible (UV–vis) spectrophotometry and PL spectroscopy. UV–vis absorption and transmittance spectra were recorded with a Lambda 750 spectrophotometer (PerkinElmer, Shelton, CT, USA) equipped with an integrating sphere. Spectra were acquired over the 400–800 nm range, covering both the 450 nm blue excitation band and the red QD emission band. PL spectra of the films were obtained using a FluoroMax Plus spectrofluorometer (Horiba, Kyoto, Japan) equipped with an integrating sphere and a 150 W xenon lamp as the excitation source. Because the film emission is collected in an integrating sphere, the measurement geometry strongly reduces the influence of the angular distribution on the detected total flux. Changes in the angular distribution introduced by TiO_2_ are therefore expected to have a limited effect on the integrated PL signal compared with a fixed angle collection geometry. Excitation was set to 450 nm to match the blue light excitation condition used in micro/mini-LED applications. The PL spectra of dilute solutions prepared from the QD ink formulations were measured using a QE-2000 system (Otsuka Electronics, Osaka, Japan) equipped with an integrating sphere. All photoluminescence measurements were performed at room temperature so the extracted self-absorption probabilities are room temperature values. The emission spectra from both the FluoroMax Plus and the QE-2000 were corrected for the spectral response of each instrument so that the film and solution spectra share a common radiometric basis for the spectral scaling described in Section 2.3. Film thicknesses were measured using a stylus profilometer (Alpha-Step D-500, KLA, Milpitas, CA, USA).

### 2.3. Extraction of the Self-Absorption Probability

**Figure 1:**
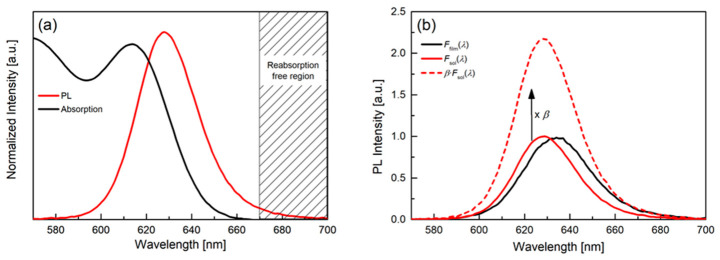
Procedure for extracting the self-absorption probability from PL spectra, following the method of Ahn et al. [20]. (**a**) Normalized absorption and PL spectra of the red CdSe/ZnSe/ZnS QDs in dilute solution. The hatched interval (670–700 nm), where absorption is negligible, is the reabsorption-free reference region. (**b**) Film PL spectrum *F*_film_(*λ*) (black) overlaid with the intrinsic emission spectrum *F*_sol_(*λ*) measured from the dilute QD solution (red solid) and the scaled form *β*·*F*_sol_(*λ*) (red dashed). The intrinsic spectrum is scaled by the factor *β* (arrow) so that *β*·*F*_sol_(*λ*) matches *F*_film_(*λ*) over the reference region using Equation (1), and the enclosed area between the two, normalized by the unattenuated emission ∫*β*·*F*_sol_(*λ*)d*λ*, gives the self-absorption probability through Equation (2).

The self-absorption probability, *a*, was directly extracted from the PL spectra using the self-absorption correction method originally developed by Ahn et al. for solid-state films [20]. This method exploits the wavelength region beyond the absorption edge, where reabsorption is negligible and the emission therefore retains its intrinsic line shape. Matching the intrinsic spectrum to the film emission in this region reconstructs the spectrum the film would emit without self-absorption. The difference between this reconstructed spectrum and the measured emission then gives the reabsorbed fraction. The full mathematical derivation of the working equations and their underlying physical assumptions are presented in the original work of Ahn et al. [20], and the present section summarizes only the procedural steps required for their application to QDCC films. The procedure consists of three steps, which are illustrated in Figure 1.

**Identification of the reabsorption-free region.** Figure 1a shows the normalized absorption and PL spectra of the red CdSe/ZnSe/ZnS QDs in dilute solution, where self-absorption is negligible owing to the low optical density. The absorption coefficient decreases sharply on the long-wavelength side of the emission band and becomes negligible relative to its in-band maximum beyond ∼660 nm, while the PL retains measurable intensity up to ∼700 nm. The interval 670–700 nm was therefore selected as the reabsorption-free reference region. This choice is further supported by considering where absorption and emission overlap. Above the absorption edge this overlap vanishes, so the 670–700 nm region carries the intrinsic emission with negligible reabsorption, whereas a window extending below the edge would include emission that is partly reabsorbed. This behavior is shown quantitatively in Figure S1. In this region, reabsorption is expected to have a minimal effect on the emission line shape, allowing the dilute solution spectrum to be scaled to the film spectrum for the self-absorption correction.

**Spectral scaling (*β*-matching).** Figure 1b shows the observed film PL spectrum *F*_film_(*λ*), measured from a spin-coated QDCC film, together with the intrinsic emission spectrum *F*_sol_(*λ*) measured from the dilute QD solution. Spectral scaling relies on a scaling factor that is determined in the reabsorption-free reference region. This factor is then applied across the whole emission band to bring the two spectra onto a common intensity scale. This allows them to be compared on a shape-only basis, independent of absolute intensity differences arising from sample geometry, QD concentration, and instrumental configuration. The scaling factor *β*, defined by Equation (1), makes the scaled intrinsic spectrum *β*·*F*_sol_(*λ*) (red dashed line) match *F*_film_(*λ*) in the reabsorption-free reference region. The subscript ‘ref’ denotes integration over this region, which was set to 670–700 nm in this study.

(1) $$\beta =\frac{\int_{ref}F_{film}\left(\lambda \right)d\lambda }{\int_{ref}F_{sol}\left(\lambda \right)d\lambda }$$

The direction of this scaling follows the method of Ahn et al. The dilute solution is the only reference that is free of self-absorption, so its spectrum is scaled to match the film over the reference region. Because *a* is a dimensionless ratio, it does not depend on the direction of scaling. After scaling, *β*·*F*_sol_(*λ*) and *F*_film_(*λ*) coincide within the reference region, as shown in Figure 1b, confirming that the long-wavelength tails of the two spectra share the same line shape and that any remaining spectral difference at shorter wavelengths can be primarily attributed to self-absorption. The film matrix could alter the emission shape in the reference region, thereby affecting the scaling. We therefore tested this in the thick, high-QD-loading films (40 wt% QD, with and without 5 wt% TiO_2_), where self-absorption is largest, using the dilute QD solution as the reference. After area normalization over the reference region, the film tails show no measurable redshift or broadening relative to the dilute solution (Figure S2). In our films, therefore, the matrix does not measurably alter the shape of the reference region, even under the most demanding conditions.

**Calculation of the self-absorption probability.** Once *β* is fixed, *β*·*F*_sol_(*λ*) represents the spectrum that the film would emit in the absence of self-absorption, while *F*_film_(*λ*) represents the spectrum actually observed. The area under the *β*·*F*_sol_(*λ*) curve is the total photon flux that would be emitted in the absence of reabsorption, and the area under the *F*_film_(*λ*) curve is the photon flux that actually escapes the film. The spectral reduction relative to the scaled intrinsic spectrum reflects the effective self-absorption experienced by the emitted photons before escape. Normalizing this reduction by the unattenuated emission yields the self-absorption probability, as given in Equation (2).

(2) $$a=1-\frac{\int_{em}F_{film}\left(\lambda \right)d\lambda }{\int_{em}\beta \cdot F_{sol}\left(\lambda \right)d\lambda }=\frac{\int_{em}\beta \cdot F_{sol}\left(\lambda \right)d\lambda -\int_{em}F_{film}\left(\lambda \right)d\lambda }{\int_{em}\beta {\cdot F}_{sol}\left(\lambda \right)d\lambda }$$

The same normalization is expressed in Figure 1b as the difference between the *β*·*F*_sol_(*λ*) and *F*_film_(*λ*) curves at short wavelengths. The integration domain “em” denotes the QD emission band and must fully encompass it. In this study, “em” was set to 570–700 nm. Physically, *a* represents the probability that a photon emitted by a QD is reabsorbed by a neighboring QD before escaping the film. A value of *a* = 0 corresponds to no reabsorption, in which case, the two areas coincide. A value of *a* close to 1 corresponds to nearly complete reabsorption.

## 3. Results

### 3.1. PL Spectra and Self-Absorption Extraction

**Figure 2:**
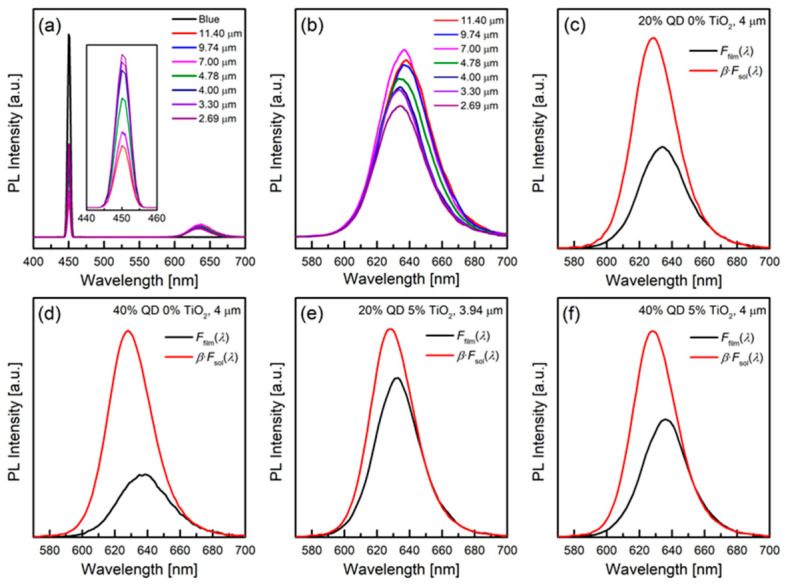
PL spectra of the QDCC films and application of the self-absorption extraction. (**a**) Full-range and (**b**) magnified red-emission PL spectra of the 20 wt% QD films without TiO_2_ under 450 nm excitation. In (**a**), the curve labeled ‘Blue’ is the incident excitation measured without a film. The inset in (**a**) magnifies the 440–460 nm region and shows the decrease in the residual blue peak with increasing film thickness. (**c**–**f**) Film emission *F*_film_(*λ*) (black) overlaid with the scaled intrinsic spectrum *β*·*F*_sol_(*λ*) (red) for representative ∼4 µm films: (**c**) 20 wt% QD, 0 wt% TiO_2_, 4 µm; (**d**) 40 wt% QD, 0 wt% TiO_2_, 4 µm; (**e**) 20 wt% QD, 5 wt% TiO_2_, 3.94 µm; (**f**) 40 wt% QD, 5 wt% TiO_2_, 4 µm.

Figure 2a,b shows the PL spectra of the 20 wt% QD films without TiO_2_, measured under 450 nm excitation at film thicknesses ranging from 2.69 to 11.4 µm. The full-range spectra in Figure 2a display two distinct bands. The residual blue peak at 450 nm corresponds to the fraction of excitation light transmitted without conversion, and the converted emission is observed in the red region. As the film thickness increases, the residual blue band diminishes while the converted red emission increases, reflecting the increasing fraction of blue photons absorbed and converted by the QDs along the optical path. This decrease is shown in the inset of Figure 2a and is quantified by the blue absorption efficiency in Section 3.3. The magnified red-emission region in Figure 2b further reveals that the emission peak shifts toward longer wavelengths as the film becomes thicker. This redshift is consistent with self-absorption within the film, as quantified in Section 3.2. The dilute QD solution, where self-absorption is negligible, emits at 628 nm, and all film peaks lie at longer wavelengths. Similar PL spectra were observed for the remaining three conditions. However, in the case of the 40 wt% films, the residual blue band showed little change with film thickness or TiO_2_ incorporation, indicating that the blue absorption efficiency was already high and only weakly dependent on thickness. The corresponding PL spectra over the full thickness range for the other conditions are provided in Figure S3 and support these observations.

Figure 2c–f illustrates the application of the self-absorption extraction procedure of Figure 1 to representative films of each of the four conditions, namely 20 and 40 wt% QD with 0 and 5 wt% TiO_2_. The films were selected to have comparable thicknesses of approximately 4 µm so that the influence of QD loading and TiO_2_ content could be compared independently of film thickness. In each panel, the observed film emission *F*_film_(*λ*) (black) is overlaid with the scaled intrinsic spectrum *β*·*F*_sol_(*λ*) (red), which was obtained as described in Section 2.3. The two curves overlap throughout the reference region for all four conditions and diverge only at shorter wavelengths, confirming that the short-wavelength deficit reflects self-absorption. The enclosed area grows with QD concentration and decreases with TiO_2_ incorporation, providing a direct spectrum-level view of how composition affects self-absorption before the quantitative analysis presented below.

### 3.2. Thickness and QD/TiO_2_ Loading Dependence of the Self-Absorption Probability

**Figure 3:**
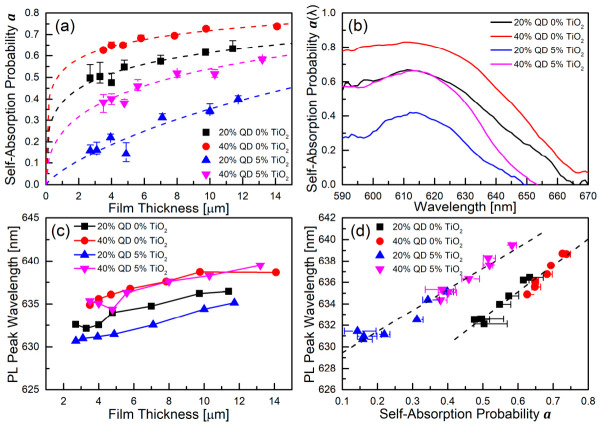
Self-absorption probability and the converted emission peak wavelength. (**a**) Self-absorption probability versus film thickness. (**b**) Wavelength-resolved self-absorption probability for the representative films of Figure 2c–f. (**c**) Converted PL peak wavelength versus film thickness. (**d**) PL peak wavelength versus self-absorption probability. Dashed curves in (**a**) are visual guides. The dashed lines in (**d**) are linear fits over the measured range, with R^2^ > 0.95 for both TiO_2_ conditions. The error bars in (**a**,**d**) represent the variation in *a* under a ±5 nm shift of the reference region boundaries.

Figure 3a shows the self-absorption probability *a* as a function of film thickness for the four conditions. For each condition, the self-absorption probability tends to increase as the film thickness increases. This trend is consistent with the fact that a thicker film provides a longer path for an emitted photon to be reabsorbed. At a given thickness, the self-absorption probability is higher for the 40 wt% films than for the 20 wt% films, because the higher QD concentration increases the absorption of the re-emitted photons. The addition of 5 wt% TiO_2_ reduces the self-absorption probability over the entire thickness range for both QD concentrations, and this reduction is larger for the 20 wt% films than for the 40 wt% films. The vertical error bars in Figure 3a show the variation in *a* when the reference region is shifted by ±5 nm, that is, to 665–695 nm and 675–705 nm. This shift changes the absolute value of *a* only modestly. The monotonic increase in *a* with thickness and the ordering among the four conditions both remain unchanged under this shift; thus, the extracted trends do not depend on the precise reference region.

Figure 3b presents the self-absorption probability resolved by wavelength for the same four films compared in Figure 2c–f. Instead of integrating over the whole emission spectrum as in Equation (2), the same ratio was evaluated at each wavelength so that the probability of reabsorption could be tracked across the emission spectrum. In all cases, the self-absorption probability is largest on the short-wavelength side of the emission and decreases toward longer wavelengths, following the wavelength dependence of the absorption coefficient. Photons emitted on the short-wavelength side of the emission spectrum fall within the absorption-emission overlap and are readily reabsorbed, whereas photons on the long-wavelength side lie beyond the absorption edge and escape with little reabsorption. The addition of 5 wt% TiO_2_ shifts the entire *a*(*λ*) curve downward. The long-wavelength side, where the self-absorption is already small, drops close to zero, while the short-wavelength side remains high. The residual self-absorption is therefore concentrated on the short-wavelength side. At each TiO_2_ condition, the gap between the two QD concentrations is most pronounced on the short-wavelength side and narrows toward longer wavelengths. This follows from the wavelength dependence of the absorption coefficient, which is high on the short-wavelength side and falls toward longer wavelengths. Where absorption is strong, raising the QD concentration adds substantial reabsorption, whereas at longer wavelengths, *a*(*λ*) approaches zero regardless of concentration.

Figure 3c shows the converted PL peak wavelength as a function of film thickness. The peak shifts toward longer wavelengths as the film thickens across all four conditions, in line with the red emission behavior observed in Figure 2b. This trend reflects the progressive accumulation of self-absorption with increasing optical path length in the film. Within each TiO_2_ condition, the peak occurs at a longer wavelength for the higher QD concentration, consistent with stronger self-absorption. At a fixed QD concentration, the two TiO_2_ conditions differ in self-absorption but show only a small difference in peak wavelength. The 40 wt% films show nearly coincident peaks, while the 20 wt% TiO_2_-containing peak lies slightly below the TiO_2_-free one.

Figure 3d replots the peak wavelength directly against the self-absorption probability. The data fall on clear linear trends, and the peak wavelength of the converted emission increases with the self-absorption probability. Within each TiO_2_ condition, the two QD concentrations follow a single line (R^2^ > 0.95 in both cases) so the relation is set by the presence or absence of TiO_2_ rather than by QD concentration. At the same integrated self-absorption probability *a*, the TiO_2_-containing films emit at a slightly longer peak wavelength. This indicates that the peak position is set not only by the amount of self-absorption but also by its spectral distribution. As shown in Figure 3b, TiO_2_ reduces *a*(*λ*) across the emission band and drives the already weak long-wavelength component close to zero, leaving the residual self-absorption concentrated toward shorter wavelengths.

### 3.3. Thickness and QD/TiO_2_ Loading Dependence of the Conversion Efficiency

**Figure 4:**
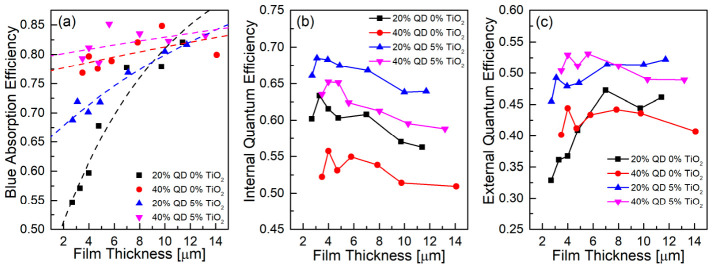
Conversion efficiency of the QDCC films versus film thickness for the four conditions. (**a**) Blue absorption efficiency *η*_abs_. (**b**) Internal quantum efficiency. (**c**) External quantum efficiency. Dashed curves in (**a**) are visual guides.

Figure 4a shows the blue absorption efficiency *η*_abs_ as a function of film thickness. Here, *η*_abs_ is the fraction of the incident blue light absorbed by the QDs. It was obtained from the integrating sphere PL measurement as the ratio of the absorbed blue intensity to the incident blue intensity measured without the film. For the 20 wt% films, *η*_abs_ is low in the thinnest films and increases as the films grow thicker. At this lower QD concentration, the incident blue light is only partially absorbed in a thin film and is absorbed more completely in a thicker one. For the 40 wt% films, *η*_abs_ is already high in the thinnest films and changes little with thickness because the higher QD concentration absorbs most of the blue light even over the short path of a thin film. The addition of 5 wt% TiO_2_ raises *η*_abs_ at a given thickness for both QD concentrations. Scattering by the TiO_2_ particles lengthens the effective path of the blue light and increases its absorption by the QDs.

Figure 4b shows the internal quantum efficiency (IQE), defined as the ratio of emitted red photons to absorbed blue photons, which was obtained from the same integrating-sphere PL measurement. In all conditions, the IQE decreases gradually as the film thickness increases. This decrease follows the thickness-dependent self-absorption probability shown in Figure 3a since a larger fraction of the converted red photons is reabsorbed and lost in thicker films. The self-absorption probability also accounts for the ordering among the four conditions. The 40 wt% films, which show the highest self-absorption in Figure 3a, exhibit the lowest IQE, whereas the addition of 5 wt% TiO_2_, which reduces the self-absorption probability, raises the IQE for both QD concentrations. The 20 wt% films with 5 wt% TiO_2_, which have the lowest self-absorption among the four conditions, accordingly show the highest IQE.

Figure 4c shows the external quantum efficiency (EQE). The EQE is the ratio of the emitted red photons, as collected by the integrating sphere, to the incident blue photons. The EQE here is an optical conversion efficiency, defined for photons rather than for electrical carriers. Light that does not escape the film, whether lost to reabsorption or trapped by total internal reflection, is excluded from this quantity by construction. It combines the two preceding quantities as their product (EQE = *η*_abs_ × IQE) since the incident blue light is first absorbed with efficiency *η*_abs_ and the absorbed light is then converted with efficiency IQE. As a result, the thickness dependence of the EQE reflects the competition between the increasing *η*_abs_ and the decreasing IQE. For the 20 wt% films, *η*_abs_ continues to increase over the measured thickness range, and this gain compensates for the gradual decrease in IQE, resulting in an overall increase in EQE, with the TiO_2_-containing film approaching a plateau after the initial rise. For the 40 wt% films, *η*_abs_ is already high and changes little with thickness so the increase of *η*_abs_ can no longer compensate for the decrease in IQE. Consequently, the EQE no longer increases with thickness and remains in a similar range across the measured thickness, in contrast to the steady rise seen for the 20 wt% films. The addition of 5 wt% TiO_2_ raises the EQE for both QD concentrations, reflecting the simultaneous increase in *η*_abs_ and IQE seen in Figure 4a,b.

## 4. Discussion

### 4.1. Determination of the Intrinsic Quantum Yield and the Role of Self-Absorption

The self-absorption probability extracted in the preceding section provides the basis for determining the intrinsic quantum yield QY_0_ of the QDs, that is, the radiative efficiency that the QDs would exhibit in the absence of any reabsorption. The relationship between the measured IQE and this intrinsic quantum yield QY_0_ is from the model of successive reabsorption and re-emission introduced by Ahn et al. [20]. A converted photon is first emitted with quantum yield QY_0_ following the absorption of a blue photon. With probability *a,* it is then reabsorbed by a neighboring QD and re-emitted, again with quantum yield QY_0_, and this sequence repeats. Summing this series of successive reabsorption and re-emission cycles yields the ratio of emitted red photons to absorbed blue photons. This ratio is the IQE of the film and corresponds to the observed quantum yield QY_obs_ of Ahn et al. [20]:

(3) $$IQE=\frac{{QY}_{0}\left(1-a\right)}{1-a\cdot {QY}_{0}}$$

Equation (3) expresses the measurable IQE in terms of the two underlying quantities QY_0_ and *a*. Solving it for QY_0_ allows the intrinsic value to be obtained from the measured IQE and self-absorption probability:

(4) $${QY}_{0}=\frac{IQE}{1-a+a\cdot IQE}$$

For each film, *a* and IQE were obtained as described in Section 3.2 and Section 3.3, respectively. Substituting these two measured quantities into Equation (4) yields QY_0_ for that film.

**Figure 5:**
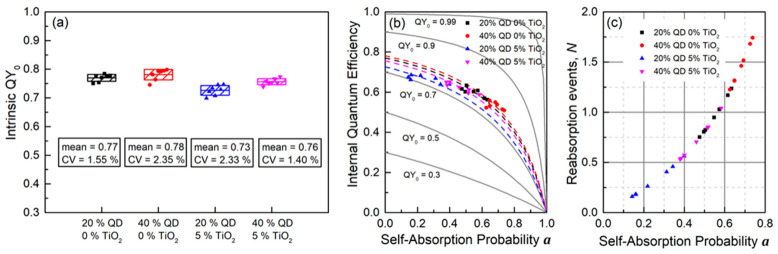
Intrinsic quantum yield, internal quantum efficiency, and the reabsorption cascade. (**a**) Intrinsic quantum yield QY_0_ determined from Equation (4) for all films, grouped by condition. (**b**) Measured IQE together with the family of curves from Equation (3), each giving the IQE expected as a function of the self-absorption probability for a fixed QY_0_, from QY_0_ = 0.3 to 0.99. The colored dashed curves are Equation (3) evaluated at the mean QY_0_ of each condition from (**a**). The measured points of each condition fall on the dashed curve of the same color. (**c**) Average number of reabsorption events per converted photon, *N*, calculated from Equation (5) using the measured self-absorption probability and QY_0_. The horizontal guideline at *N* = 1 marks the transition from less than one to more than one reabsorption event per converted photon on average.

Figure 5a shows the QY_0_ values determined in this way for all films, grouped by condition. Within each condition, QY_0_ is nearly constant across the full thickness range, with a coefficient of variation (the ratio of the standard deviation to the mean) of only 1.4 to 2.4%. This constancy provides the central validation of the approach. The inputs to Equation (4) vary strongly with thickness since the IQE decreases and the self-absorption probability increases as the film grows thicker, yet the extracted QY_0_ does not. Because QY_0_ is an intrinsic property of the QDs, this thickness independence indicates that the correction consistently removes the reabsorption contribution. Because QY_0_ here refers to the QDs within the cured film, its value reflects the radiative efficiency in that environment rather than in dilute solution. The extraction is therefore supported by its internal consistency across thickness rather than against an external standard. At the high QD loading of 40 wt%, Förster resonance energy transfer (FRET) between neighboring QDs may also contribute since it is a near-field, nonradiative channel favored by short interparticle distances. However, the way the self-absorption probability is extracted here limits the influence of FRET on it. This process depends on the QD concentration and interparticle spacing, which are held fixed within each condition, while the self-absorption probability is obtained by only varying the film thickness at a fixed concentration. Within a given thickness series, the FRET contribution can therefore be regarded as essentially constant, so the observed thickness dependence of the self-absorption probability is more naturally attributed to far-field reabsorption. Any reduction in the intrinsic quantum yield associated with FRET would be largely thickness independent and would be absorbed into QY_0_. Taken together with the redshift that grows with thickness, this constancy of QY_0_ suggests that the thickness dependence of the IQE is governed mainly by far-field reabsorption.

Figure 5b shows the measured IQE of the QD films together with the family of curves given by Equation (3), where each curve gives the IQE expected as a function of the self-absorption probability for a fixed value of QY_0_. The curves range from QY_0_ = 0.3 at the bottom to QY_0_ = 0.99 at the top. At *a* = 0, where no reabsorption occurs, each curve starts at IQE = QY_0_, so the curve meets the vertical axis at its own QY_0_ value. Along each curve, the IQE decreases as the self-absorption probability increases since each reabsorption carries a chance of nonradiative loss and a larger fraction of the converted photons is ultimately lost. Two features of these curves are relevant to the present films.

First, a higher QY_0_ maintains a high IQE even when the self-absorption probability is substantial. At a given self-absorption probability, the IQE is larger for QDs with a higher QY_0_, because a reabsorbed photon is then more likely to be re-emitted (with probability QY_0_) than lost (with probability 1 − QY_0_) at each reabsorption event. This contrast is clear from the curves. On the QY_0_ = 0.3 curve, the IQE drops to low values as soon as the self-absorption probability becomes moderate. In contrast, on the QY_0_ = 0.99 curve, a reabsorbed photon is almost always re-emitted rather than lost so the IQE stays high across the same range of self-absorption probability. The QDs studied here have QY_0_ values between 0.73 and 0.78 and therefore lie on the upper curves of the family, which is favorable for maintaining a high IQE.

Second, the decrease in the IQE becomes steeper as the self-absorption probability increases. Along any curve, a given increase in the self-absorption probability lowers the IQE by a larger amount when the self-absorption probability is already high. This steepening reflects the reabsorption cascade that underlies Equation (3). As the self-absorption probability rises, a re-emitted photon is more likely to be reabsorbed again, so each further increase removes a larger share of the converted light.

This steepening is visible in the thickness behavior of the individual conditions. The 40 wt% films without TiO_2_ lie at high self-absorption probabilities (0.63 to 0.74, Figure 3a), where the curves are steep. Although their self-absorption probability varies over only a narrow range with thickness, their IQE changes by an amount comparable to that of the other conditions so that the change in IQE per unit change in self-absorption probability is the largest among the four conditions. The 20 wt% films with 5 wt% TiO_2_ show the opposite case. They lie at low self-absorption probabilities, from 0.14 to 0.40, where the curves are less steep, so that even a wide variation in self-absorption probability with thickness produces only a moderate change in IQE.

Taken together, the four conditions fall along the curve family in the order set by their self-absorption probability. This diagram places the IQE of every film within a common framework defined by QY_0_ and the self-absorption probability, and makes explicit how these two quantities together determine the IQE of each condition.

Figure 5c shows the average number of reabsorption events that a converted photon undergoes before it leaves the film or is lost, calculated from the measured self-absorption probability and QY_0_. We count reabsorption events rather than successful re-emissions, because each reabsorption is the point at which both nonradiative loss and spectral shift occur, regardless of whether the photon is subsequently re-emitted.

The number of reabsorption events is based on the same reabsorption and re-emission cascade that underlies Equation (3). After a converted photon is emitted, it passes through a series of independent steps, and in each step, one of three outcomes occurs. The photon escapes the film without reabsorption, with probability 1 − *a*. It is reabsorbed with probability *a* and then re-emitted with probability QY_0_, giving a combined probability *a*·QY_0_, in which case the process continues to a further step. Alternatively, it is reabsorbed with probability *a* but lost without re-emission with probability 1 − QY_0_, giving *a*(1 − QY_0_). The process only continues when the photon is reabsorbed and re-emitted, so the probability that it terminates at any given step is *q* = (1 − *a*) + *a*(1 − QY_0_) = 1 − *a*·QY_0_. Because each step terminates with the same probability *q* independently of the preceding ones, the number of steps until termination follows a geometric distribution, whose mean is 1/*q*. A reabsorption occurs in a step only when the photon is reabsorbed, with probability *a*, so each step contributes *a* reabsorption on average. The average number of reabsorption events is therefore the mean number of steps, 1/*q*, multiplied by *a*:

(5) $$N=\frac{a}{1-a\cdot {QY}_{0}}$$

The denominator of Equation (5) controls how *N* grows with the self-absorption probability. When the self-absorption probability is small, *a*·QY_0_ is far below unity, the denominator is close to one, and *N* increases almost linearly. As the self-absorption probability increases, *a*·QY_0_ approaches unity, the denominator becomes small, and *N* rises steeply. The quantity *a*·QY_0_ is the probability that an emitted photon is reabsorbed and then re-emitted, which returns it to the cascade for a further step. When this probability is small, most photons escape after at most one reabsorption. When it grows large, a photon is reabsorbed and re-emitted several times before it finally escapes or is lost. This feedback is what makes *N* rise steeply at high self-absorption probability.

A useful reference point on this curve is *N* = 1, which marks the transition from a regime where a typical photon is reabsorbed at most once to one where it is reabsorbed more than once on average. The 20 wt% films with 5 wt% TiO_2_, with the lowest self-absorption probabilities, remain well below this point, undergoing on average less than one reabsorption per photon. The 40 wt% films without TiO_2_, with the highest self-absorption probabilities, lie above this point, undergoing on average more than one reabsorption per photon.

The number of reabsorption events connects the trends of the preceding sections. The increase in *N* with the self-absorption probability is reflected in both the decrease in IQE in Figure 4 and the redshift in Figure 3. Each reabsorption carries a chance of nonradiative loss and preferentially removes short-wavelength photons, lowering the IQE and shifting the spectrum toward longer wavelengths. Both effects are therefore governed by the self-absorption probability, with *N* measuring how often reabsorption occurs.

### 4.2. The Role of TiO_2_ in Photon Management

The two optical effects of TiO_2_ observed here (the increase in the blue absorption efficiency and the reduction in the self-absorption probability) can be understood together from the different angular distributions of the incident and converted light. The blue excitation light enters the film as a nearly collimated beam close to the surface normal. Scattering by the TiO_2_ particles deflects this beam and lengthens its optical path within the film, increasing the probability that it is absorbed by the QDs before transmission, as reflected in the higher blue absorption efficiency *η*_abs_ in Figure 4a. The converted red light, in contrast, is emitted isotropically by the QDs [26]. A large fraction is emitted beyond the escape cone and is trapped within the film by total internal reflection, where it undergoes repeated reabsorption. Scattering redirects part of this trapped light into the escape cone and allows it to leave the film before further reabsorption. Scattering can also send part of the light along longer paths within the film, where it is more likely to be reabsorbed before escaping. These two effects compete. Because the EQE measured in the integrating sphere counts only the photons that actually escape the film in any direction, the net outcome is captured directly in the data. The EQE increases with TiO_2_ for both concentrations (Figure 4c), which indicates that the gain from redirection outweighs the added reabsorption loss under the present conditions. The higher blue absorption efficiency and the higher IQE therefore reinforce each other, giving the TiO_2_-containing films the highest EQE in Figure 4c. This collimated condition is also the geometry used to obtain the reported values. Under the more Lambertian excitation of an operating device, the oblique components of the incident light lengthen the average optical path through the film and increase the blue absorption. The self-absorption probability reported here is therefore a quantity specific to the film and measured under this collimated geometry. Its absolute value would shift under device excitation, but the relative effects of concentration, thickness, and TiO_2_ are expected to persist.

The same redirection also reshapes the self-absorption spectrum. Because the redirection does not depend strongly on the emission wavelength, it lowers *a*(*λ*) across the emission band. The wavelength dependence of the scattering is set by the particle size relative to the wavelength. The 200–300 nm rutile particles are of the same order as the emission wavelength in the film and therefore lie in the Mie regime, in which the strong *λ*^−4^ wavelength dependence of scattering by small particles in the Rayleigh regime no longer applies. Across the narrow emission band, the redirection is therefore nearly independent of wavelength. Since the self-absorption is already small on the long-wavelength side, *a*(*λ*) there is driven close to zero. The residual self-absorption therefore concentrates toward shorter wavelengths because of the asymmetry that *a*(*λ*) already has, not because the scattering selects particular wavelengths (Figure 3b). This short-wavelength concentration of the residual self-absorption accounts for the larger peak shift of the TiO_2_-containing films at the same self-absorption probability in Figure 3d.

The angular redistribution of the converted light is a general consequence of scattering by dielectric particles and is not specific to the particle size used here. According to classical light scattering theory, the scattering strength and angular distribution depend on the particle size relative to the wavelength and on the refractive index contrast [27]. Particles much smaller than the wavelength fall in the Rayleigh or Rayleigh-like regime and scatter weakly, whereas particles much larger than the wavelength scatter strongly but tend to redirect the light more in the forward direction. The 200–300 nm particles used here fall between these limits, comparable to the emission wavelength in the film, so appreciable angular redistribution of the trapped light is expected. Identifying the optimal particle size would require a separate scattering or ray tracing analysis.

## 5. Conclusions

Self-absorption in QDCC films has long been recognized as a key factor, but has been assessed only through indirect indicators. In this work, we showed that the self-absorption probability can be directly extracted from PL spectra using the spectral correction method of Ahn et al. The reliability of this extraction is supported by the intrinsic quantum yield from the same analysis, which remains independent of film thickness within each condition.

This quantitative extraction yields both the integrated self-absorption and its wavelength-resolved profile. The integrated self-absorption and the intrinsic quantum yield together account for the IQE. The IQE and the blue absorption efficiency then combine to give the EQE, including its thickness dependence. The wavelength-resolved profile explains the peak wavelength of the converted emission. Adding TiO_2_ lowers the extracted self-absorption probability and concentrates its residue toward shorter wavelengths through light scattering. Because the peak wavelength depends on both the amount and the spectral distribution of the self-absorption, the two TiO_2_ conditions follow distinct relations between peak wavelength and self-absorption probability.

Several directions remain for future work. The extraction method demonstrated here is not inherently limited to Cd-based QDs and may be extended to cadmium-free QDs and other color-conversion systems, provided that an appropriate reabsorption-free reference region can be identified. Extending the measurement to device-level excitation, where the incident light is more Lambertian, would connect the film-specific self-absorption probability reported here to operating conditions. The role of self-absorption could be probed further using time-resolved photoluminescence, which would directly track reabsorption and re-emission dynamics, and using temperature-dependent measurements, which would reveal how the self-absorption probability responds to thermal shifts in the absorption and emission spectra. Finally, a dedicated scattering or ray-tracing analysis, together with a direct measurement of the angular emission profile by goniophotometry, would identify the particle size that most effectively redirects the trapped light and would quantify the angular redistribution introduced by TiO_2_.

By quantifying self-absorption as an extractable parameter, this approach provides a practical design descriptor for QDCC films, linking conversion efficiency and emission wavelength to the same underlying reabsorption process.

## Data Availability

The original contributions presented in this study are included in the article/Supplementary Materials. Further inquiries can be directed to the corresponding author.

## Supplementary Materials

The following supporting information can be downloaded at: https://www.mdpi.com/article/10.3390/nano16140842/s1, Figure S1: Extracted self-absorption probability *a* as a function of the lower bound of the reference region, for the four conditions; Figure S2: Area-normalized photoluminescence spectra in the 670–700 nm reference region for the QD dilute solution and for the thick films of the two 40 wt% QD conditions; Figure S3: Photoluminescence spectra of the QD color-conversion films over the full thickness range for three conditions; Table S1: Spin-coating speed and the corresponding film thickness for each condition.
