# Supplementary material for: Quantitative Extraction of the Self-Absorption Probability in Quantum Dot Color Conversion Films and Its Modulation by TiO2
Source: Nanomaterials (Basel). 2026 Jul 9;16(14):842. doi: 10.3390/nano16140842 (PMC13414827; doi:10.3390/nano16140842)
Supplement: Supplementary file 1 [file nanomaterials-16-00842-s001.zip › nanomaterials-4413775-supplementary.pdf]

nanomaterials

MDPI

Supplementary Information

Quantitative extraction of the self-absorption probability in quantum dot color conversion films and its modulation by TiO_2

Kinza Batool [1], Youngji Lim [2], Kyoungwon Park [2] and Bum-Joo Lee [1,*]

[1] Department of Flexible and Printable Electronics, Jeonbuk National University, 567, Baekje-daero, Deokjin-gu, Jeonju-si 54896, Republic of Korea
[2] Display Research Center, Korea Electronics Technology Institute (KETI), 25, Saenari-ro, Bundang-gu, Seongnam-si 13509, Republic of Korea
* Correspondence: bumjoolee@jbnu.ac.kr

Table S1. Spin-coating speed and the corresponding film thickness for each condition. Thicknesses were measured by stylus profilometry.

Condition	RPM	Thickness (μm)
20 wt% QD, 0 wt% TiO_2	8000	2.69
	6800	3.30
	5000	4.00
	3400	4.78
	1900	7.00
	1200	9.74
	1000	11.40
40 wt% QD, 0 wt% TiO_2	8000	3.50
	6800	4.00
	5500	4.70
	3814	5.80
	2253	7.85
	1495	9.77
	1000	14.10
20 wt% QD, 5 wt% TiO_2	8000	2.70
	6800	3.10
	5000	3.94
	3400	4.89
	1900	7.10
	1200	10.00
	1000	11.73
40 wt% QD, 5 wt% TiO_2	8000	3.50
	6800	4.00
	5500	4.78
	3814	5.60
	2253	8.00
	1495	10.30
	1068	13.20

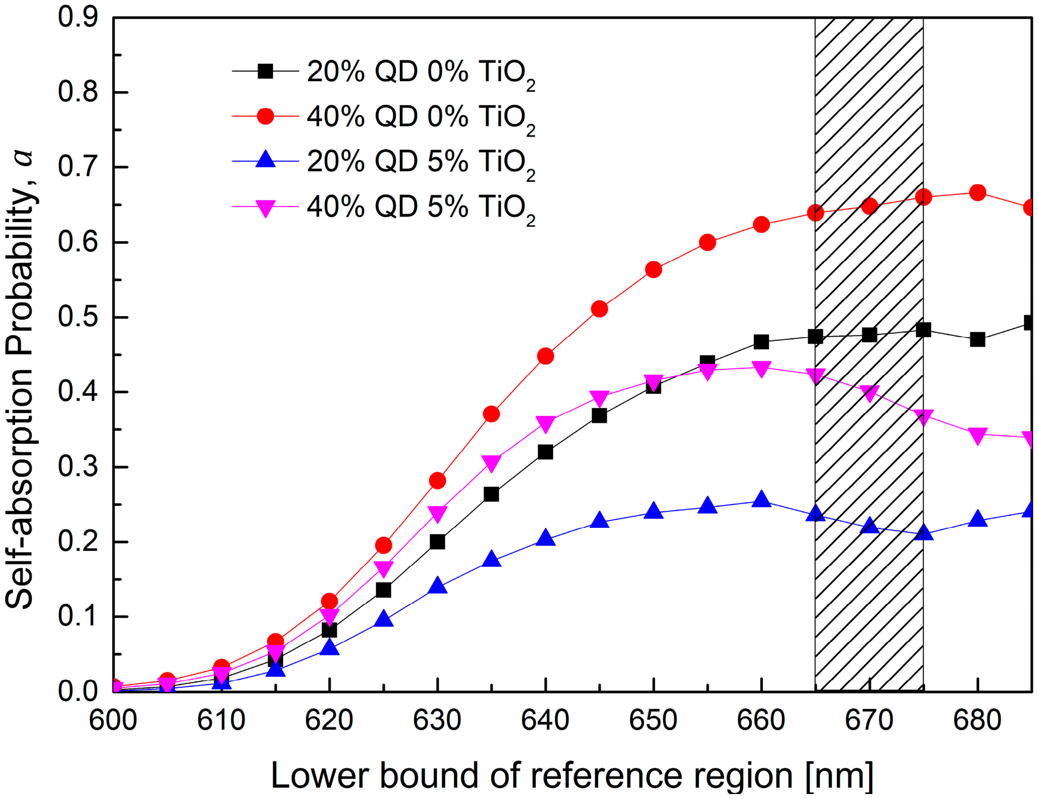

Figure S1. Extracted self-absorption probability *a* as a function of the lower bound of the reference region, for the four conditions (20 and 40 wt% QD, without and with 5 wt% TiO_2) at a film thickness of ~4 μm. The lower bound was varied from 600 to 685 nm while the upper bound was fixed at 700 nm. For lower bounds below ~650 nm, *a* decreases steeply because the reference region enters the absorption–emission overlap, where the film emission is itself reduced by reabsorption; beyond the absorption edge (~660 nm), *a* approaches a plateau and becomes much less sensitive to the exact lower bound. The hatched band (665–675 nm) marks the ±5 nm range around the chosen lower bound of 670 nm, which lies on this plateau. The plateau is less flat for the TiO_2-containing conditions, most noticeably for the film with 40 wt% QD and 5 wt% TiO_2, where *a* shows a more pronounced variation across the reference region. Nonetheless, within the ±5 nm band the ordering among the four conditions is preserved — *a* increases with QD concentration and decreases with TiO_2 addition — indicating that the extracted trends do not depend on the precise choice of the reference region.

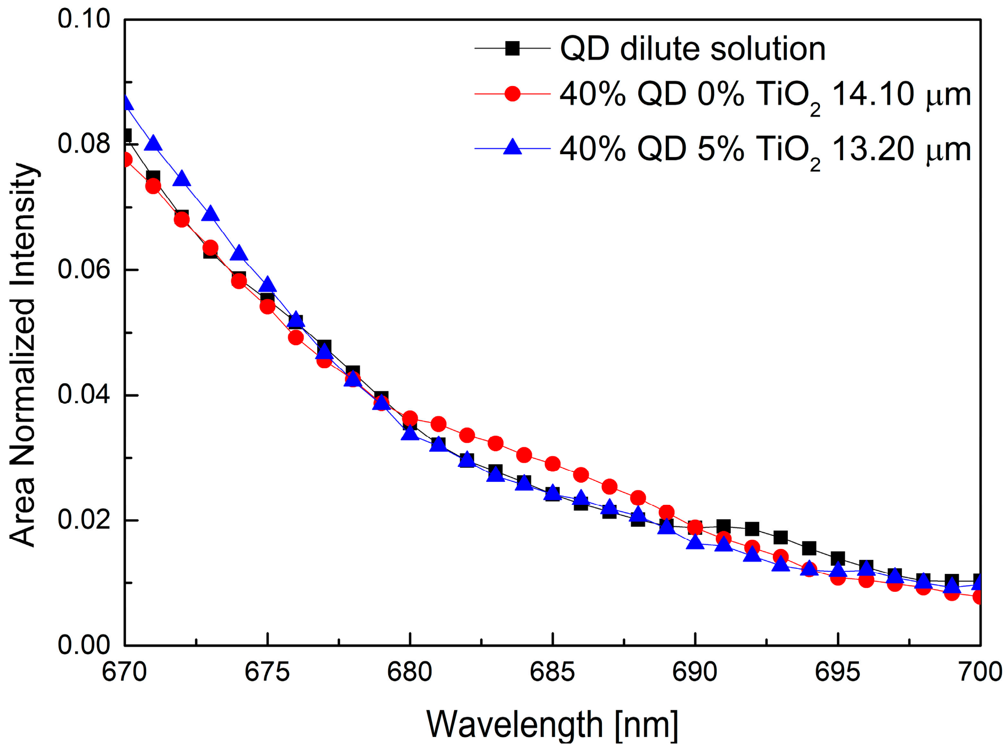

Figure S2. Area-normalized photoluminescence spectra in the 670–700 nm reference region for the QD dilute solution and for the thick films of the two 40 wt% QD conditions, without and with 5 wt% TiO_2 (14.10 and 13.20 μm, the highest-thickness specimens). The dilute solution is the intrinsic, reabsorption-free reference. These thick, high-concentration films correspond to the largest self-absorption in the sample set. After area normalization, the three spectra overlap closely and show no systematic redshift or broadening. To quantify the peak position of the tail, the intensity-weighted mean wavelength (spectral centroid) over the reference region was calculated as $\lambda_c = \Sigma\lambda_i{\cdot}I_i\ /\ \Sigma I_i$, where I_i is the intensity at wavelength λ_i. The centroids are 679.7 nm (solution), 679.6 nm (0% TiO_2 film), and 679.2 nm (5% TiO_2 film), agreeing to within ~0.5 nm. This indicates that the reference-region tail shape in the films is essentially the same as the intrinsic emission and is consistent with negligible reabsorption in the 670–700 nm region. All spectra were measured at room temperature.

https://doi.org/10.3390/nano16140842

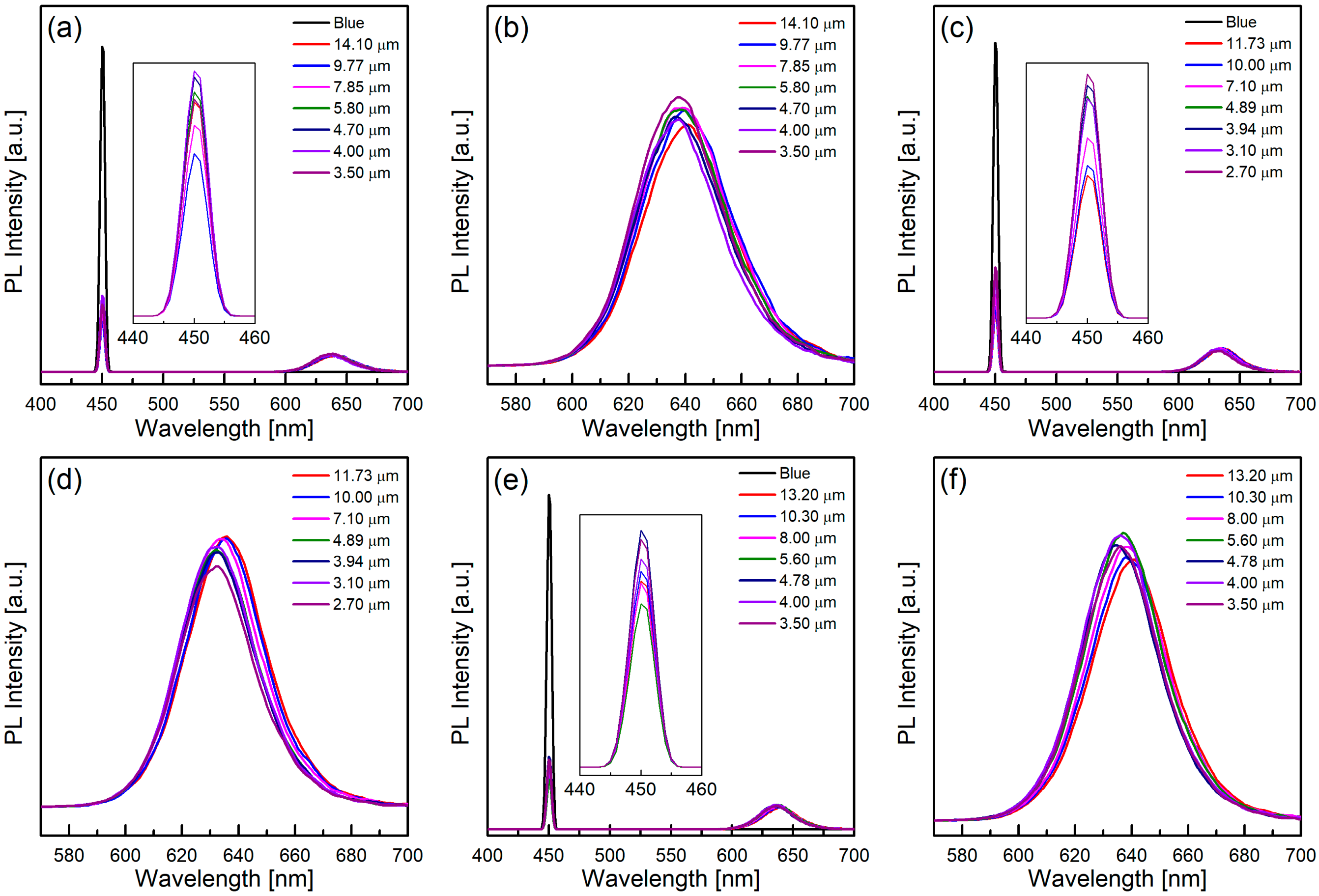

Figure S3. Photoluminescence (PL) spectra of the QD color-conversion films over the full thickness range for three conditions. (**a**,**b**) 40 wt% QD without TiO_2; (**c**,**d**) 20 wt% QD with 5 wt% TiO_2; (**e**,**f**) 40 wt% QD with 5 wt% TiO_2. The two panels of each pair (full-range and expanded red-emission) show the same films over different spectral windows: (**a**,**c**,**e**) the full range (400–700 nm), including the residual unconverted blue light near 450 nm and the converted red emission near 640 nm; (**b**,**d**,**f**) the converted red emission on an expanded scale. The insets in (**a**,**c**,**e**) magnify the residual unconverted blue light near 450 nm (the incident blue excitation is omitted from the insets so that the thickness dependence of the residual blue is visible). In each panel, the curves correspond to different film thicknesses as indicated in the legend, and "Blue" denotes the incident blue excitation.

Disclaimer/Publisher's Note: The statements, opinions and data contained in all publications are solely those of the individual author(s) and contributor(s) and not of MDPI and/or the editor(s). MDPI and/or the editor(s) disclaim responsibility for any injury to people or property resulting from any ideas, methods, instructions or products referred to in the content.

https://doi.org/10.3390/nano16140842
